# Expressions of MMPs and TIMP-1 in Gastric Ulcers May Differentiate *H. pylori*-Infected from NSAID-Related Ulcers

**DOI:** 10.1100/2012/539316

**Published:** 2012-05-01

**Authors:** Hsiu-Chi Cheng, Hsiao-Bai Yang, Wei-Lun Chang, Wei-Ying Chen, Yi-Chun Yeh, Bor-Shyang Sheu

**Affiliations:** ^1^Institute of Clinical Medicine, College of Medicine, National Cheng Kung University, 138 Sheng Li Road, Tainan 70403, Taiwan; ^2^Department of Internal Medicine, National Cheng Kung University Hospital, College of Medicine, National Cheng Kung University, 138 Sheng Li Road, Tainan 70403, Taiwan; ^3^Department of Pathology, National Cheng Kung University Hospital, College of Medicine, National Cheng Kung University, 138 Sheng Li Road, Tainan 70403, Taiwan; ^4^Department of Pathology, Ton-Yen General Hospital, 69 Xian Zheng 2nd Road, Zhubei, Hsinchu 30268, Taiwan; ^5^Institute of Basic Medical Sciences, College of Medicine, National Cheng Kung University, 138 Sheng Li Road, Tainan 70403, Taiwan

## Abstract

*Background*. Two major causes of gastric ulcers are *Helicobacter pylori* (*H. pylori*) infection and nonsteroidal anti-inflammatory drug (NSAID) use. *Aims*. This study aimed to determine if there were different expressions of matrix metalloproteinases (MMPs) and tissue inhibitor of matrix metalloproteinase-1 (TIMP-1) between *H. pylori*-infected and NSAID-related ulcers. *Methods*. The 126 gastric ulcer patients (*H. pylori* infected *n* = 46; NSAID related *n* = 30; combined with two factors *n* = 50) provided ulcer and nonulcer tissues for assessment of MMP-3, -7, and -9 and TIMP-1 expression by immunohistochemical staining. *Results*. Gastric ulcer tissues had significantly higher MMP-3, -7, and -9 and TIMP-1 expressions than nonulcer tissues (*P* < 0.05). *H. pylori*-infected gastric ulcers had even higher MMP-7, MMP-9, and TIMP-1 expressions in epithelial cells than NSAID-related gastric ulcers (*P* < 0.05). In patients with the two combined factors, gastric ulcers expressed similar proportions of antral ulcers and MMP-7 and MMP-9 intensities to NSAID-related gastric ulcers, but lower MMP-9 and TIMP-1 than *H. pylori*-infected gastric ulcers (*P* < 0.05). *Conclusions. H. pylori*-infected gastric ulcers express higher MMP-7, MMP-9, and TIMP-1 than NSAID-related ulcers. In patients with the two combined factors, ulcer location and MMP-7 and MMP-9 intensities are similar to NSAID use.

## 1. Introduction

*Helicobacter pylori* (*H. pylori*) infection and nonsteroidal anti-inflammatory drug (NSAID) use are the two most important causes of gastric ulcers but their clinical courses are somewhat different [1]. In non-NSAID users, *H. pylori* eradication enhances peptic ulcer healing [2]. However, among NSAID users, gastric ulcer healing becomes slower in *H. pylori-*eradicated or negative patients than in *H. pylori*-infected patients [3, 4]. This implies that there should be different local expression of molecules involved in gastric ulcer healing between *H. pylori*-infected and NSAID-related cases.

Gastric ulcer healing is assisted by the development of granulation tissues and remodeling at the ulcer base [5]. Matrix metalloproteinases (MMPs) play major roles in ulcer tissue remodeling and are inhibited by tissue-derived inhibitors like the tissue inhibitor of matrix metalloproteinases (TIMPs) [6, 7]. Recently, several studies have shown that MMP expression can be regulated by either *H. pylori *infection or by NSAID use [8–18]. However, these data are mostly derived from animal settings, and human clinical data remains rare, especially in assessing MMP expression in gastric ulcers among patients with combined *H. pylori* infection and NSAID use.

Accordingly, this prospective study aimed to determine if specific MMP and TIMP expressions are upregulated in gastric ulcer tissues and if there are different MMP expressions among patients with *H. pylori* infection, NSAID use, or both *in vivo* (in human gastric tissues). The study also aimed to determine if MMP-9 and TIMP-1 expression levels in gastric ulcers could be rational markers for identifying patients who could potentially benefit from *H. pylori* eradication without interference or accelerating gastric ulcer healing in patients with combined *H. pylori* infection and NSAID use.

## 2. Methods

### 2.1. Patients and Study Design

The hospital's research and ethics committee approved the study design, and informed consent was obtained from all participants prior to enrollment. Patients who received upper gastroscopy for melena, hematochezia, hematemesis, or dyspepsia, and those with active gastric ulcers, were identified and consecutively enrolled. Patients were excluded if they had tumor or ulcer bleeding because of mechanical factors (i.e., gastrostomy tube induction), bleeding diathesis, or hematological disorders, such as idiopathic thrombocytopenia, aspirin use, clopidogrel, warfarin, or cyclooxygenase-2 selective inhibitors (i.e., celecoxib, etoricoxib). Because of etiologic heterogeneity, patients who were neither *H. pylori* infected nor NSAID users were also excluded.

Gastric mucosal biopsies were obtained under direct vision gastroscopy (GIF-XQ 260 Endoscope, Olympus Medical Systems Co., Ltd, Tokyo, Japan) using standard biopsy forceps (Olympus FB-25K-1). Eight biopsies were taken during each gastroscopic session, including six from the edge of ulcer tissues and two from the nonulcerated antral mucosa at least 2 cm from the ulcer as nonulcer tissues [19].

Patients were diagnosed with *H. pylori* infection either by positive rapid urease test (CLO test, Kimberly-Clark, Draper, Utah, USA) or histologically [19, 20]. The *H. pylori*-infected group was defined as having positive test for *H. pylori* and without history of NSAID, aspirin, analgesic, or traditional Chinese medicine use within four weeks before enrollment. The NSAID-related group was defined as continuous or sporadic use of nonselective NSAIDs more than three times per week during the past four weeks and a negative test for *H. pylori*. The combined *H. pylori*-infection and NSAID-use group was defined as having a positive test for *H. pylori* and a history of NSAID use defined as the NSAID-related group.

### 2.2. Immunohistochemistry Studies for Gastric MMP and TIMP-1 Expressions

Tissue immunohistochemical staining was performed using mouse monoclonal antibodies of anti-human-MMP-3, -7, -9, or TIMP-1 (Chemicon International, Inc., Temecula, CA, USA). The gastric tissue was fixed in 10% buffered formalin, embedded in paraffin, and serially sectioned at 4 *μ*m thickness. To stop endogenous peroxidase activity, the specimen was immersed for 20 mins in 3% hydrogen peroxide and then pretreated with Dako Cytomation Target Retrieval Solution (Dako, Carpinteria, CA, USA) for antigen retrieval. The nonspecific binding sites were saturated with diluted normal blocking serum.

The tissue section was treated with primary antibody against MMP-3, -7, -9 or TIMP-1 at a dilution of 1 : 1000 and then incubated overnight in a humidified chamber at 4°C. The Vectastain Elite ABC Kit (Vector Laboratories, Inc., Burlingame, CA, USA) was used for blocking, linkage, and labeling for staining according to the manufacturer's instructions. The Dako Cytomation Liquid DAB + Substrate Chromogen system was used as chromogen. The section was then counterstained with hematoxylin. Colon ulcer tissue was used as positive control.

The same pathologist blinded to patients' background scored the staining of MMPs and TIMP-1. The expression grades of MMP-7, MMP-9, and TIMP-1 in the superficial epithelium or MMP-3 and TIMP-1 in inflammatory cells of the lamina propria were scored as the percentage of positive-stained cells. The score ranged from 0–4, as 0 (negative), 1 (<5% cells), 2 (5%–29% cells), 3 (30%–59% cells), and 4 (≥60% cells) as before [21, 22]. Otherwise, MMP-9 expression in inflammatory cells of the lamina propria was scored as 0 (no expression), 1 (<5 cells/high power field [HPF]), 2 (5–10 cells/HPF), 3 (11–20 cells/HPF), and 4 (>20 cells/HPF).

### 2.3. Statistical Analysis

The Pearson's  *χ* ^2^  test and one-way analysis of variance (ANOVA) were used to compare multiple groups, while Tukey's least significant difference test was used to identify statistically significant groups. The Wilcoxon signed-rank test was used to compare the differences of MMP-3, -7, and -9 or TIMP-1 expression between “ulcer tissues” and “nonulcer tissues”. The Mann-Whitney *U* test was applied to assess MMP or TIMP-1 intensity in gastric tissues between different study groups. The diagnostic value of MMP-9 and TIMP-1 expression in gastric ulcer tissues for detecting *H. pylori* infection was measured using the following three criteria: sensitivity, specificity, and likelihood ratios. The receiver operating characteristic (ROC) curves for determining MMP and TIMP-1 expression cut-off values that best discriminated between *H. pylori*-infected and NSAID-related gastric ulcers were derived by plotting the sensitivity versus 1 minus the specificity for each MMP and TIMP-1 expression value. The optimal cut-off point was defined as the closest point on the receiver operating characteristic curve to the point at a 1 minus specificity of zero and a sensitivity of 100%. All of the tests were two-tailed and the statistical significance was defined as *P* < 0.05.

## 3. Results

### 3.1. Demographic Features of the Study Patients

The study prospectively identified 126 (50 women and 76 men) patients with active gastric ulcers. Their mean age was 65.4 years. The gastric ulcer groups included 46 (36.5%) as *H. pylori*-related infections, 30 (23.8%) as NSAID users, and 50 (39.7%) with combined *H. pylori* infection and NSAID exposure. There was a significant difference in ulcer location and female proportion between the three study groups (Table 1). The* H. pylori*-infected group had higher corpus ulcer tendency than the other two groups (34.8% versus 3.3% or 8.0%, resp., *P* = 0.002). The NSAID-related group had higher female proportion (56.7% versus 23.9% or 44.0%, *P* = 0.01). Otherwise, there was no difference in the demographic background and other clinical characteristics among the three gastric ulcer groups (*P* > 0.05).

### 3.2. Higher MMP-3, -7, and -9 and TIMP-1 Expressions in Ulcer Tissues

**Figure 1:**
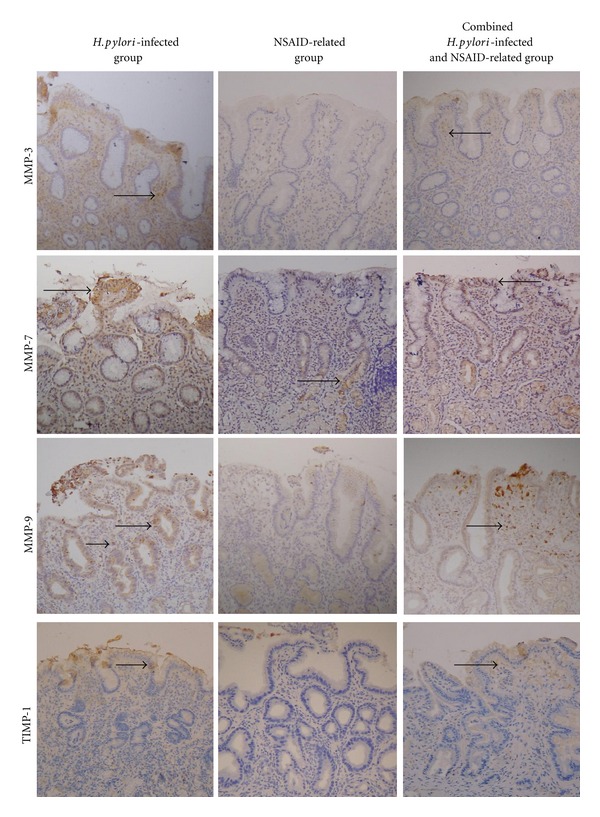
The immunohistochemical stains for matrix metalloproteinase (MMP)-3, -7, and -9 and tissue inhibitor of matrix metalloproteinase (TIMP)-1 in the *Helicobacter pylori- *(*H. pylori-*) infected group, in the nonsteroidal anti-inflammatory drug- (NSAID-) related group, and in the combined *H. pylori*-infection and NSAID-use group, in superficial epithelial cells and inflammatory cells of the lamina propria of gastric tissues (600x). *H. pylori-*infected gastric ulcers express higher MMP-7, MMP-9, and TIMP-1 than NSAID-related ulcers. Arrows indicate positive staining.

**Figure 2:**
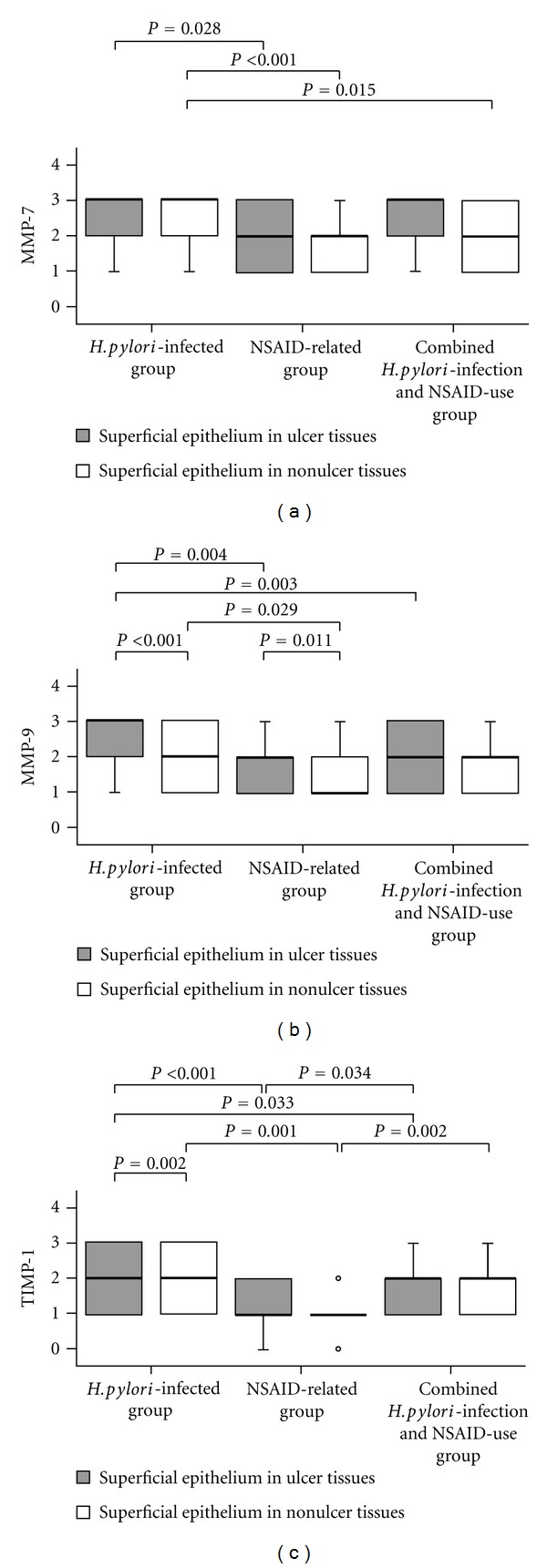
Box plots of the distributions and median scores of MMP-7, and -9 and TIMP-1 in the superficial epithelium of gastric ulcer tissues and nonulcer tissues in different groups, respectively. The *P* values compared scores according to patients in the *H. pylori*-infected, NSAID-related, and combined *H. pylori*-infection and NSAID-use groups by Mann-Whitney *U* test, and also compared scores according to ulcer tissues and nonulcer tissues by Wilcoxon signed-rank test. In the box plots, the 75th and 25th percentiles are represented by the top and bottom of the box, respectively. The horizontal lines refer to the medians. Abbreviations are as in Figure 1.

**Figure 3:**
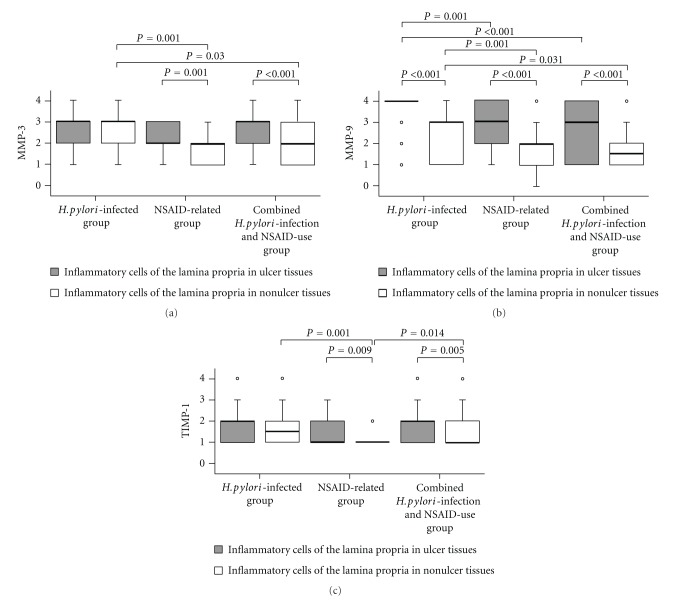
Box plots of the distributions and median scores of MMP-3, MMP-9, and TIMP-1 in inflammatory cells of the lamina propria of gastric ulcer tissues and nonulcer tissues in different groups, respectively. The *P* values compared scores according to patients in the *H. pylori*-infected, NSAID-related, and combined *H. pylori*-infection and NSAID-use groups by Mann-Whitney *U* test and also compared scores according to ulcer tissues and nonulcer tissues by Wilcoxon signed-rank test. In the box plots, the 75th and 25th percentiles are represented by the top and bottom of the box, respectively. The horizontal lines refer to the medians. Abbreviations are as in Figure 1.

Over gastric tissues, MMP-7, MMP-9, and TIMP-1 were positively stained in epithelial cells and MMP-3, MMP-9, and TIMP-1 in inflammatory cells of the lamina propria (Figure 1). Moreover, in the *H. pylori*-infected group, MMP-9 and TIMP-1 expressions over the superficial epithelium (Figure 2) and MMP-9 expression over inflammatory cells of the lamina propria (Figure 3) of gastric ulcer tissues were higher than in nonulcer tissues, respectively. In the NSAID-related group, MMP-9 expression over the superficial epithelium of ulcer tissues was higher than that in nonulcer tissues (Figure 2). Moreover, in the NSAID-related group and the combined *H. pylori*-infection and NSAID-use group, MMP-3, MMP-9, and TIMP-1 expression over inflammatory cells of the lamina propria of ulcer tissues were higher than in nonulcer tissues (Figure 3) (*P* < 0.05).

MMP-7, MMP-9, and TIMP-1 expressions over the superficial epithelium of gastric ulcer tissues were higher than in nonulcer tissues (*P* < 0.05). MMP-3, MMP-9, and TIMP-1 expressions over inflammatory cells of the gastric ulcer lamina propria were also higher than in nonulcer tissues (*P* < 0.001) (Table 2).

### 3.3. Different MMP and TIMP-1 Expressions over Gastric Tissues among the Study Groups

In nonulcer tissues, MMP-3, -7, and -9 and TIMP-1 expressions were significantly lower in the NSAID-related group than in* H. pylori*-infected group, either over the superficial epithelium or inflammatory cells of the lamina propria (*P* < 0.05) (Figures 2 and 3). The MMP and TIMP-1 expressions in the combined *H. pylori*-infection and NSAID-use group were between the other groups.

Over superficial epithelium of gastric ulcer tissues, MMP-7, MMP-9, and TIMP-1 expressions were significantly lower in the NSAID-related group than in the* H. pylori*-infected group (*P* < 0.05). Moreover, MMP-9 and TIMP-1 expressions were lower in the combined *H. pylori*-infection and NSAID-use group than in the* H. pylori*-infected group (*P* < 0.05). However, there were similar MMP-7 and MMP-9 expressions between the* H. pylori*-infected NSAID users and the noninfected NSAID users (*P* > 0.05) (Figure 2). Over inflammatory cells of the gastric ulcer lamina propria, only MMP-9 expression (not MMP-3 and TIMP-1) was significantly higher in the* H. pylori*-infected group than in the other two NSAID-use groups with or without *H. pylori *infection (*P* < 0.001) (Figure 3).

In ulcer tissues, MMP and TIMP-1 expressions were similar between different gender or between different ulcer locations (Female versus male in the superficial epithelium: MMP-7, *P* = 0.46; MMP-9, *P* = 0.11; TIMP-1, *P* = 0.34; in inflammatory cells of the lamina propria: MMP-3, *P* = 0.96; MMP-9, *P* = 0.60; TIMP-1, *P* = 0.26; Antrum versus corpus versus both in the superficial epithelium: MMP-7, *P* = 0.23; MMP-9, *P* = 0.30; TIMP-1, *P* = 0.21; in inflammatory cells of the lamina propria: MMP-3, *P* = 0.64; MMP-9, *P* = 0.58; TIMP-1, *P* = 0.41).

### 3.4. Prediction of Ulcer Etiology in the Combined *H. pylori*-Infected and NSAID-Use Group

The cut-off values assessed by ROC curves to define the optimal diagnostic accuracy of “*H. pylori*-infected” ulcer were 2.5 for MMP-9 (area of ROC curve [AUC] 0.68, 95% CI: 0.56–0.81, *P* = 0.007) and 1.5 for TIMP-1 (AUC 0.74, 95% CI: 0.63–0.85, *P* = 0.001) over gastric ulcer epithelial cells, respectively. The cut-off point as 3.5 of MMP-9 over the inflammatory cells of the gastric ulcer lamina propria also achieved significant AUC as 0.69 (95% CI: 0.56–0.81, *P* = 0.007). Gastric ulcer tissues with a combined pattern of MMP-9 ≥3 and TIMP-1 ≥2 in ulcer epithelial cells and MMP-9 ≥ 4 in the ulcer inflammatory cells significantly defined the “*H. pylori-*related ulcer pattern” (AUC 0.67, 95% CI: 0.55–0.79, *P* = 0.011). Only 10% (5/50) of ulcer tissues of *H. pylori*-infected NSAID users expressed an “*H. pylori*-related ulcer pattern”.

## 4. Discussion

This study demonstrates that MMP-3, -7 and -9 and TIMP-1 may play a potential role in gastric ulcer formation or the healing process. The findings indicate that *H. pylori-*infected gastric ulcers express higher MMP-7, MMP-9, and TIMP-1 compared to NSAID-related ulcers. Moreover, in patients with these two combined factors, MMP-7 and MMP-9 intensities are similar to NSAID use. The data is particularly important as it supports the hypothesis that ulcer characteristics in *H. pylori*-infected NSAID users are mainly similar to NSAID exposure rather than *H. pylori* infection. Accordingly, this study supports the tentative clinical suggestion of treating gastric ulcer first for NSAID users, irrespective to the presence of *H. pylori *infection.

In human stomachs, MMPs and TIMPs are expressed normally or are upregulated during epithelial regeneration or tissue remodeling [9, 23–26]. By comparing gastric ulcer and nonulcer tissues within the same individual, this study is highly original in revealing how MMP-3, -7, and -9 and TIMP-1 expressions are upregulated in gastric ulcers induced by *H. pylori* infection and NSAID use, respectively (*P* < 0.05; Table 2, Figures 2 and 3). Because of the dual roles of MMPs and TIMPs in the process of either ulcer formation or healing, it is important to study MMP and TIMP expressions in different gastric ulcer etiology.

Previous studies have shown that *H. pylori* infections induce MMP-7 and MMP-9 expressions via activation of nuclear factor kappa light-chain enhancer of activated B cells (NF-*κ*B) [15]. However, NSAIDs inhibit tumor necrosis factor- (TNF-) induced NF-*κ*B activation [27]. Because NF-*κ*B and MMPs play a relevant role in the processes of ulcer healing, blocking NF-*κ*B activation may result in impaired ulcer repair [28]. *H. pylori* infection is also associated with upregulation of TIMP-1 [18]. The current study validates that MMP-7, MMP-9, and TIMP-1 expressions are significantly higher in *H. pylori*-infected patients than in NSAID users (*P* < 0.05) (Figures 2 and 3). These data partly explain why longer healing is required in the NSAID-related ulcers than in the *H. pylori*-infected ulcers [15].

TIMP-1 is expressed strongly in epithelial cells and weakly in inflammatory cells in *H. pylori*-infected gastric mucosa, but absent in uninfected subjects [18]. In our study, Figures 2 and 3 showed that in nonulcer tissue, the *H. pylori*-infected group has higher TIMP-1 expression than in the NSAID-related group either in the superficial epithelium or in inflammatory cells (*P* = 0.001). However, in ulcer tissues, inflammatory cells had higher TIMP-1 upregulation than in nonulcer tissues, especially in the NSAID-related group and in the combined *H. pylori*-infection and NSAID-use group (*P* = 0.009 and 0.005, resp., Figure 3). TIMP-1 is an endogenous MMP inhibitor produced by the same cells which express MMPs [9, 26]. In addition, the upregulation of MMP-3 in inflammatory cells of ulcer tissues is similar to TIMP-1 (Figure 3). We proposed that inflammatory cells of gastric ulcers participate vigorously in the healing process and upregulate MMP-3 and TIMP-1 strongly either in* H. pylori* infection or in the other two NSAID-use groups with or without *H. pylori *infection (Table 2).

Due to different expressions of MMPs and TIMP-1 between *H. pylori*-infected and NSAID-related gastric ulcers, cut-off values of MMPs and TIMP-1 have been measured to discriminate the possible characteristics: *H. pylori *infection or NSAID use? The results show stronger patterns of MMP-9 and TIMP-1 expression in *H. pylori*-infected gastric ulcer tissues. As such, in most (45/50, 90%) patients with combined *H. pylori*-infection and NSAID-use ulcers, it is possible that NSAID-induced gastropathy rather than *H. pylori* infection is the principal characteristic. In addition, the current study also shows that *H. pylori*-infected gastric ulcers have corpus predilection compared to NSAID-related ulcers with or without *H. pylori* infection. This is compatible with findings by Al-Assi et al. (Table 1) [29].

Our results showed that combined *H. pylori*-infection and NSAID-use ulcers are similar to NSAID-related ulcers but are different from *H. pylori*-infected ulcers. The reason why combined *H. pylori*-infection and NSAID-use ulcers were similar to NSAID-induced gastropathy cannot be fully ascertained. We proposed that NSAIDs inhibit TNF-induced NF-*κ*B activation and downregulate MMP-7 and MMP-9 expressions induced by *H. pylori* infection. Nevertheless, this may be the reason why eradicating *H. pylori* does not enhance gastric ulcer healing in such patients [3, 4]. Based on the translation evidence of this study, treating such gastric ulcers with proton-pump inhibitors first may not eradicate *H. pylori* before ulcer healing but result in *H. pylori*-induced cyclooxygenase-2 and MMP-9 upregulation. This may also potentiate the inhibition effect of omeprazole on gastric acid secretion [15, 28, 30–32].

Previous study showed MMP-1 concentration is significantly higher in *H. pylori*-induced ulcers compared to NSAID-induced ones [33]. Our study further validated the difference of MMP-9 and TIMP-1 expression on the ulcer etiology. More importantly, our study showed that the possible major cause of ulcerogenesis in combined *H. pylori*-infection and NSAID-use ulcers is favored to be NSAID-related rather than *H. pylori* induced. In this study, we did not investigate MMP-2 and TIMP-2 expression because MMP-2 in *H. pylori* infection and indomethacin treatment has limited significance [15, 17, 34, 35]. Moreover, TIMP-2 appears to be designed specifically to interact with MMP-2 [36]. On the other hand, it will be interested to compare MMP and TIMP-1 expressions in the middle or final stage of the healing process between *H. pylori-*infected ulcers and NSAID-related ulcers.

In summary, *H. pylori-*infected gastric ulcers express higher MMP-9 and TIMP-1 than NSAID-related ulcers. Based on the predominant ulcer location over the antrum and the weaker MMP-9 and TIMP-1 expressions in gastric ulcer tissues, gastric ulcers in *H. pylori-*infected NSAID users may be predominantly similar to those in NSAID use. As such, ulcer healing should be the first management objective, followed by *H. pylori* later, in cases of ulcers concurrent with *H. pylori *infection and NSAID exposure.

## Figures and Tables

**Table 1:** Demographic and clinical characteristics of the three gastric ulcer groups.

Ulcer groups				
Parameters	*H. pylori *infected (*n* = 46)	NSAID related (*n* = 30)	Combined *H. pylori *infection and NSAID use (*n* = 50)	*P* value
Mean age (years)	62.2 (16.6)	64.1 (14.4)	69.2 (13.1)	0.06^†^
Women (%)	23.9	56.7	44.0	0.01^‡^
Size of ulcer (cm)	1.4 (1.0)	1.3 (1.1)	1.7 (1.5)	0.20^†^
Number of ulcers (*n*), single : multiple	18 : 28	7 : 23	12 : 38	0.19^‡^
Ulcer locations (*n*) antrum : corpus : both	21 : 16 : 9	21 : 1 : 8	33 : 4 : 13	0.002^‡^
Hemoglobin (g/dL)	10.9 (2.8)	9.5 (2.9)	10.1 (2.9)	0.13^†^
Albumin (g/dL)	3.5 (0.8)	3.7 (0.8)	3.5 (0.6)	0.60^†^
Creatinine (mg/dL)	1.5 (1.6)	1.6 (1.9)	1.3 (1.1)	0.69^†^

^†^Mean (SD or standard deviation), by one-way ANOVA test.

^‡^The chi-square test. Normal range, Hemoglobin 13.5–17 g/dL; Albumin 3–5 g/dL; Creatinine 0.7–1.5 mg/dL.

*H. pylori*: *Helicobacter pylori*; NSAID: Nonsteroidal anti-inflammatory drugs.

**Table 2:** The distributions of MMP-3, -7, and -9 and TIMP-1 net over different histologic locations of gastric ulcer tissues compared to non-ulcer tissues.

Net increase (*n*, %)	Positive	Equal	Negative	*P * ^†^ value
Superficial epithelium				
MMP-7	35 (27.8%)	70 (55.5%)	21 (16.7%)	0.023
MMP-9	49 (38.9%)	65 (51.6%)	12 (9.5%)	<0.001
TIMP-1	28 (22.2%)	87 (69.1%)	11 (8.7%)	0.005
Inflammatory cells of gastric lamina propria				
MMP-3	43 (34.1%)	76 (60.3%)	7 (5.6%)	<0.001
MMP-9	75 (60.0%)	38 (30.4%)	12 (9.6%)	<0.001
TIMP-1	32 (25.4%)	86 (68.3%)	8 (6.3%)	<0.001

^†^ *P* values indicate the significant difference of higher MMP-3, -7, and -9 and TIMP-1 intensities on ulcer tissues compared to antral non-ulcer tissues (assessed by Wilcoxon signed-rank test).

MMP: matrix metalloproteinases; TIMP: tissue inhibitor of matrix metalloproteinase.

## Abbreviations

MMP: Matrix metalloproteinase
TIMP: Tissue inhibitor of matrix metalloproteinase
NSAID: Non-steroidal anti-inflammatory drugs
*H. pylori*: *Helicobacter pylori*
HPF: High power field
ANOVA: Analysis of variance
ROC curves: Receiver operating characteristic curves
SD: Standard deviation
NF-*κ*b: Nuclear factor kappa light-chain enhancer of activated B cells.

## References

[1] Tytgat GNJ (2000). Ulcers and gastritis. *Endoscopy*.

[2] Arkkila PET, Seppälä K, Kosunen TU (2003). Eradication of *Helicobacter pylori* improves the healing rate and reduces the relapse rate of non-bleeding ulcers in patients with bleeding peptic ulcer. *American Journal of Gastroenterology*.

[3] Hawkey CJ, Tulassay Z, Szczepanski L (1998). Randomised controlled trial of *Helicobacter pylori* eradication in patients on non-steroidal anti-inflammatory drugs: HELP NSAIDs study. *The Lancet*.

[4] Bianchi Porro G, Parente F, Imbesi V, Montrone F, Caruso I (1996). Role of *Helicobacter pylori* in ulcer healing and recurrence of gastric and duodenal ulcers in longterm NSAID users. Response to omeprazole dual therapy. *Gut*.

[5] Tarnawski AS (2005). Cellular and molecular mechanisms of gastrointestinal ulcer healing. *Digestive Diseases and Sciences*.

[6] Mignatti P, Rifkin DB, Welgus HG, Parks WC, Clark RA (1996). Proteinases and tissue remodeling. *The Molecular and Cellular Biology of Wound Repair*.

[7] Birkedal-Hansen H, Moore WGI, Bodden MK (1993). Matrix metalloproteinases: a review. *Critical Reviews in Oral Biology and Medicine*.

[8] Tomita M, Ando T, Minami M (2009). Potential role for matrix metallo- proteinase-3 in gastric ulcer healing. *Digestion*.

[9] Pender SLF, MacDonald TT (2004). Matrix metalloproteinases and the gut—new roles for old enzymes. *Current Opinion in Pharmacology*.

[10] Wroblewski LE, Noble PJM, Pagliocca A (2003). Stimulation of MMP-7 (matrilysin) by *Helicobacter pylori* in human gastric epithelial cells: role in epithelial cell migration. *Journal of Cell Science*.

[11] Crawford HC, Krishna US, Israel DA, Matrisian LM, Washington MK, Peek RM (2003). *Helicobacter pylori* strain-selective induction of matrix metalloproteinase-7 *in vitro* and within gastric mucosa. *Gastroenterology*.

[12] McCaig C, Duval C, Hemers E (2006). The Role of Matrix Metalloproteinase-7 in Redefining the Gastric Microenvironment in Response to *Helicobacter pylori*. *Gastroenterology*.

[13] Bergin PJ, Anders E, Sicheng W (2004). Increase production of matrix metalloproteinases in *Helicobacter pylori*-associated human gastritis. *Helicobacter*.

[14] Lempinen M, Inkinen K, Wolff H, Ahonen J (2000). Matrix metalloproteinases 2 and 9 in indomethacin-lnduced rat gastric ulcer. *European Surgical Research*.

[15] Mori N, Sato H, Hayashibara T (2003). *Helicobacter pylori* induces matrix metalloproteinase-9 through activation of nuclear factor *κ*B. *Gastroenterology*.

[16] Salmela MT, Pender SLF, Karjalainen-Lindsberg ML, Puolakkainen P, MacDonald TT, Saarialho-Kere U (2004). Collagenase-1 (MMP-1), matrilysin-1 (MMP-7), and stromelysin-2 (MMP-10) are expressed by migrating enterocytes during intestinal wound healing. *Scandinavian Journal of Gastroenterology*.

[17] Kubben FJGM, Sier CFM, Schram M (2007). Eradication of *Helicobacter pylori* infection favourably affects altered gastric mucosal MMP-9 levels. *Helicobacter*.

[18] Bodger K, Ahmed S, Pazmany L (2008). Altered gastric corpus expression of tissue inhibitors of metalloproteinases in human and murine Helicobacter infection. *Journal of Clinical Pathology*.

[19] Sheu BS, Sheu SM, Yang HB, Huang AH, Wu JJ (2003). Host gastric Lewis expression determines the bacterial density of *Helicobacter pylori* in babA2 genopositive infection. *Gut*.

[20] Sheu BS, Lin CY, Lin XZ, Shiesh SC, Yang HB, Chen CY (1996). Long-term outcome of triple therapy in *Helicobacter pylori*-related nonulcer dyspepsia: a prospective controlled assessment. *American Journal of Gastroenterology*.

[21] Sung JJY, Leung WK, Go MYY (2000). Cyclooxygenase-2 expression in *Helicobacter pylori*-associated premalignant and malignant gastric lesions. *American Journal of Pathology*.

[22] Sheu BS, Yang HB, Sheu SM, Huang AH, Wu JJ (2003). Higher gastric cycloxygenase-2 expression and precancerous change in *Helicobacter pylori*-infected relatives of gastric cancer patients. *Clinical Cancer Research*.

[23] Tatsuguchi A, Fukuda Y, Ishizaki M, Yamanaka N (1999). Localization of matrix metalloproteinases and tissue inhibitor of metalloproteinases-2 in normal human and rabbit stomachs. *Digestion*.

[24] Saarialho-Kere UK, Vaalamo M, Puolakkainen P, Airola K, Parks WC, Karjalainen-Lindsberg ML (1996). Enhanced expression of matrilysin, collagenase, and stromelysin-1 in gastrointestinal ulcers. *American Journal of Pathology*.

[25] Schuppan D, Hahn EG (2000). MMPs in the gut: inflammation hits the matrix. *Gut*.

[26] Boone TC, Johnson MJ, De Clerck YA, Langley KE (1990). cDNA cloning and expression of a metalloproteinase inhibitor related to tissue inhibitor of metalloproteinases. *Proceedings of the National Academy of Sciences of the United States of America*.

[27] Takada Y, Bhardwaj A, Potdar P, Aggarwal BB (2004). Nonsteroidal anti-inflammatory agents differ in their ability to suppress NF-*κ*B activation, inhibition of expression of cyclooxygenase-2 and cyclin D1, and abrogation of tumor cell proliferation. *Oncogene*.

[28] Takahashi S, Fujita T, Yamamoto A (2001). Role of nuclear factor-*κ*B in gastric ulcer healing in rats. *American Journal of Physiology*.

[29] Al-Assi MT, Genta RM, Karttunen TJ, Graham DY (1996). Ulcer site and complications: relation to *Helicobacter pylori* infection and NSAID use. *Endoscopy*.

[30] Colucci R, Fornai M, Antonioli L (2009). Characterization of mechanisms underlying the effects of esomeprazole on the impairment of gastric ulcer healing with addition of NSAID treatment. *Digestive and Liver Disease*.

[31] Hudson N, Balsitis M, Filipowicz F, Hawkey CJ (1993). Effect of *Helicobacter pylori* colonisation on gastric mucosal eicosanoid synthesis in patients taking non-steroidal anti-inflammatory drugs. *Gut*.

[32] Pawlik T, Konturek PC, Konturek JW (2002). Impact of *Helicobacter pylori* and nonsteroidal anti-inflammatory drugs on gastric ulcerogenesis in experimental animals and in humans. *European Journal of Pharmacology*.

[33] Menges M, Chan CC, Zeitz M, Stallmach A (2000). Higher concentration of matrix-metalloproteinase 1 (interstitial collagenase) in *H. pylori*-compared to NSAID-induced gastric ulcers. *Zeitschrift fur Gastroenterologie*.

[34] Ito A (1995). Cyclooxygenase inhibitors augment the production of pro-matrix metalloproteinase 9 (progelatinase B) in rabbit articular chondrocytes. *FEBS Letters*.

[35] Yokoyama T, Otani Y, Kurihara N (2000). Matrix metalloproteinase expression in cultured human gastric wall fibroblasts—interactions with *Helicobacter pylori* isolated from patients with ulcers. *Alimentary Pharmacology and Therapeutics, Supplement*.

[36] Howard EW, Bullen EC, Banda MJ (1991). Regulation of the autoactivation of human 72-kDa progelatinase by tissue inhibitor of metalloproteinases-2. *The Journal of Biological Chemistry*.

